# Descriptive epidemiology of small intestinal malignancies: the German Cancer Registry experience

**DOI:** 10.1038/sj.bjc.6690541

**Published:** 1999-07

**Authors:** A Stang, C Stegmaier, B Eisinger, R Stabenow, K A Metz, K-H Jöckel

**Affiliations:** Institute of Medical Informatics, Biometry and Epidemiology, Institute of Pathology, Medical Faculty, University of Essen, Hufelandstr. 55, Essen, 45122, Germany; Saarland Cancer Registry, Statistisches Landesamt Saarland, Postfach 10 30 44, Saarbrücken, 66119, Germany; Common Cancer Registry of the Federal States of Berlin, Brandenburg, Mecklenburg-Vorpommern, Sachsen-Anhalt and the Free States of Sachsen and Thüringen, Brodauer Str. 16–22, Berlin, 12621, Germany

**Keywords:** small intestine, cancer, incidence, epidemiology, registries, Germany

## Abstract

In the first population-based analysis of certain epidemiologic features of primary malignancies of the small intestine in Germany, we used data from the Saarland Cancer Registry (1982–1993) and from the former National Cancer Registry of the German Democratic Republic (1976–1989). The age-standardized incidence rates for ages 0–74 years is 3.3–6.2 per million per year. The average incidence rates of the federal state Saarland are for men about 1.3 times and for women about 1.4 times the rate of the former German Democratic Republic. After the age of 30 years, the incidence rates increased with increasing age. Incidence rates for carcinoids levelled off after the age of 54 years. Rates for men were 35–40% higher than for women after adjusting for age. The risk for carcinomas, malignant carcinoids and malignant lymphoma were higher for men than for women. © 1999 Cancer Research Campaign


*British Journal of Cancer* (1999) **80**(9), 1440–1444
© 1999 Cancer Research Campaign
Article no. bjoc.1999.0541

### Descriptive epidemiology of small intestinal malignancies: the German Cancer Registry experience

**A Stang[1], C Stegmaier[3], B Eisinger[4], R Stabenow[4], KA Metz[2] and K-H Jöckel[1]**

[1]Institute of Medical Informatics, Biometry and Epidemiology, and [2]Institute of Pathology, Medical Faculty, University of Essen, Hufelandstr. 55, 45122 Essen, Germany; [3]Saarland Cancer Registry, Statistisches Landesamt Saarland, Postfach 10 30 44, 66119 Saarbrücken, Germany; [4]Common Cancer Registry of the Federal States of Berlin, Brandenburg, Mecklenburg-Vorpommern, Sachsen-Anhalt and the Free States of Sachsen and Thüringen, Brodauer Str. 16–22, 12621 Berlin, Germany

**Summary** In the first population-based analysis of certain epidemiologic features of primary malignancies of the small intestine in Germany, we used data from the Saarland Cancer Registry (1982–1993) and from the former National Cancer Registry of the German Democratic Republic (1976–1989). The age-standardized incidence rates for ages 0–74 years is 3.3–6.2 per million per year. The average incidence rates of the federal state Saarland are for men about 1.3 times and for women about 1.4 times the rate of the former German Democratic Republic. After the age of 30 years, the incidence rates increased with increasing age. Incidence rates for carcinoids levelled off after the age of 54 years. Rates for men were 35–40% higher than for women after adjusting for age. The risk for carcinomas, malignant carcinoids and malignant lymphoma were higher for men than for women.

**Keywords:** small intestine; cancer; incidence; epidemiology; registries; Germany

Primary malignant tumours of the small intestine (TSI) are defined as tumours that originate from the duodenum, jejunum, ileum or Meckel's diverticulum. According to the ninth revision of the International Classification of Diseases (1977), TSI are coded as 152.

The rarity of TSI is an important reason for the comparatively late interest in the epidemiologic features of these tumours. To our knowledge, the first epidemiological analysis based on population-based data was published in 1968 (Brookes et al, 1968). Population-based analyses of the epidemiology of TSI have not been published until now in the Federal Republic of Germany (FRG). At present, the Federal Republic of Germany does not have cancer registries that cover the complete geographic territory. Out of the existing cancer registries, the Saarland Cancer Registry and the National Cancer Registry of the German Democratic Republic (GDR) (until 1989) are the only registries with a data quality, i.e. completeness that is comparable with international cancer registries (Parkin et al, 1997). Therefore, the epidemiological features of malignant tumours can only be analysed by regionally limited population-based cancer registries within the FRG.

The aim of this study is to present the epidemiological characteristics of TSI in the FRG based on cancer registry data of the GDR and the federal state Saarland. Results are compared with international epidemiological findings.

### MATERIALS AND METHODS

We analysed data from the Saarland Cancer Registry (Statistisches Landesamt Saarbrücken, 1996) for the period 1982–1993 and from the GDR for the period 1976–1989. Incidence rates were standardized by using the world population as the standard (WSR) (Parkin et al, 1997). We calculated truncated incidence rates (age 0–74 years) because the registration of cancer in the elderly (i.e. 75 years and more) in the former GDR probably was incomplete due to underreporting.

Malignant lymphomas of the small intestine that are assigned to the International Classification of Diseases (ICD)-9 codes 200 or 202 'Malignant neoplasms of lymphatic and haematopoetic tissue' were included in the incidence rates. Primary extranodal malignant lymphoma of the small intestine coded with 200 or 202 were identified by comparing topography codes of the *International Classification of Diseases for Oncology* (ICD-O; Percy et al, 1990) with the corresponding ICD-O morphology codes. We only included TSI with behaviour that was coded as primary malignant (fifth digit of morphology code = 3). Only carcinoids were also included with behaviour code '1' (i.e. neoplasm of uncertain behaviour) because these tumours are also considered as malignant (except those of the appendix).

We analysed the topographical distribution of TSI only for the GDR because the information about the topography of TSI was determined in only 45% of the cases of the federal state of Saarland. We do not present the sex-specific histologic distribution of TSI from Saarland because the precision of these estimates for the Saarland Cancer Registry was too low.

Analyses of the histological distribution of the tumours were based on the ICD-O (Percy et al, 1990). Histologies coded according to the first edition of ICD-O (WHO, 1976) were transferred to codes of the second edition of ICD-O. We used a median/average smoothing process to dampen the roughness of the age-specific incidence rates for the four histopathological subgroups of the GDR so that any underlying pattern is more clearly seen (Selvin, 1996). We compared our results with data from other cancer registries published by the International Agency

*Received 24 February 1998*
*Revised 7 December 1998*
*Accepted 19 January 1999*

*Correspondence to:* A Stang

**Table 1** Sex-specific incidence rates of primary malignancies of the small intestine in the GDR (1046 cases) and the federal state Saarland (101 cases)

| Registry | Period | Population in millions (residents) | Sex | Incidence rate[a] | Cases (*n*)[b] | Male : Female ratio |
|---|---|---|---|---|---|---|
| Saarland | 1982–1993 | 1 | Males | 6.2 | 51 | 1.35 |
| | | | Females | 4.6 | 50 | |
| GDR | 1976–1989 | 17 | Males | 4.6 | 514 | 1.40 |
| | | | Females | 3.3 | 532 | |

[a]Per 1 000 000 subjects per year; age-standardized (WSR), truncated 0–74 years; [b]GDR: calendar year 1985 is excluded from the calculation of the incidence rates.

**Table 2** Incidence rates (WSR) of the histopathological subgroups of primary malignant tumours of the small intestine

| Registry | Sex | Carcinomas | | Malignant carcinoids | | Sarcomas | | Malignant lymphomas | |
|---|---|---|---|---|---|---|---|---|---|
| | | N | IR | N | IR | N | IR | N | IR |
| Saarland (1982–1993) | M + F | 43 | 2.2 | 32 | 1.7 | 12 | 0.7 | 6 | 0.3 |
| GDR (1976–1989)[a] | M + F | 350 | 1.2 | 312 | 1.1 | 172 | 0.7 | 159 | 0.6 |
| | M | 164 | 1.4 | 165 | 1.5 | 80 | 0.7 | 86 | 0.8 |
| | F | 186 | 1.1 | 147 | 0.9 | 92 | 0.6 | 73 | 0.5 |

M: males, F: females; n: number of cases, IR: age-truncated (0–74 years) and age-standardized incidence rates (world standard); cases per million. [a]GDR: calendar year 1985 is excluded from the calculation of the incidence rates.

**Table 3** Topographical and histological distribution of 1046 morphologically confirmed primary malignant tumours of the small intestine in the GDR (1976–1989)[a]

| | Duodenum | Duodenojejunal flexura | Jejunum | Ileum | Not otherwise specified | Overall |
|---|---|---|---|---|---|---|
| Carcinoma | 0.37 | 0.08 | 0.34 | 0.20 | 0.23 | 1.22 |
| Malignant carcinoid | 0.17 | 0.11 | 0.11 | 0.46 | 0.25 | 1.10 |
| Sarcoma | 0.06 | 0.02 | 0.18 | 0.21 | 0.19 | 0.65 |
| Malignant lymphoma | 0.03 | 0.02 | 0.11 | 0.23 | 0.24 | 0.62 |
| Others | 0.01 | 0.00 | 0.00 | 0.01 | 0.01 | 0.03 |
| Overall | 0.64 | 0.23 | 0.74 | 1.11 | 0.92 | |

[a]Age-standardized incidence rates (WSR), truncated 0–74 years; cases per million; calendar year 1985 is excluded from the calculation of the incidence rates; all incidence rates less than 0.005 were rounded down.

for Research on Cancer (IARC), which presents incidence data of cancer registries of the whole world in a standardized way (Parkin et al, 1992, 1997). We combined data for the registration periods 1983–1987 (Parkin et al, 1992) and 1988–1992 (Parkin et al, 1997) to facilitate the comparison with our findings and to get more stable incidence rates.

### RESULTS

Between 1976 and 1989, 1046 persons under 75 years of age of the GDR (about 17 million residents) were diagnosed with TSI. Morphologically confirmed cases reported to the registry were coded according to the ICD-O (1046 cases) with the exception of the calendar year 1985 (97 cases). Ninety-seven per cent of the cases (cases from 1985 excluded) were morphologically confirmed. The overall incidence rate for the registration periods was 4.6 per million males and 3.3 per million females per year.

In the federal state Saarland 101 cases under age of 75 years with TSI were reported to the cancer registry between 1982 and 1993. About 98% of these cases were morphologically confirmed. The overall incidence rate for the registration periods was 6.2 per million males and 4.6 per million females per year. The overall incidence rates of the federal state Saarland for men were about 1.35 times, and for women about 1.40 times, the rate of the GDR.

After the age of 30 years, the incidence of TSI increased with increasing age. Men had a higher risk of TSI then women (Saarland: 35%, GDR: 40% respectively), after adjusting for age (Table 1).

For neither cancer registry was there evidence of an increase or decrease in the incidence rates of the overall group of TSI or histopathological subgroup over time (data not shown). Table 2 presents the incidence rates of the histopathological subgroups of TSI. We found the highest risk for carcinomas and malignant carcinoids in both registries. The risk is about 45% and 35% lower for carcinomas and malignant carcinoids in the former GDR, after adjusting for age. The risk for lymphomas in the former GDR is about twice as high as compared to Saarland, after adjusting for age.

© 1999 Cancer Research Campaign

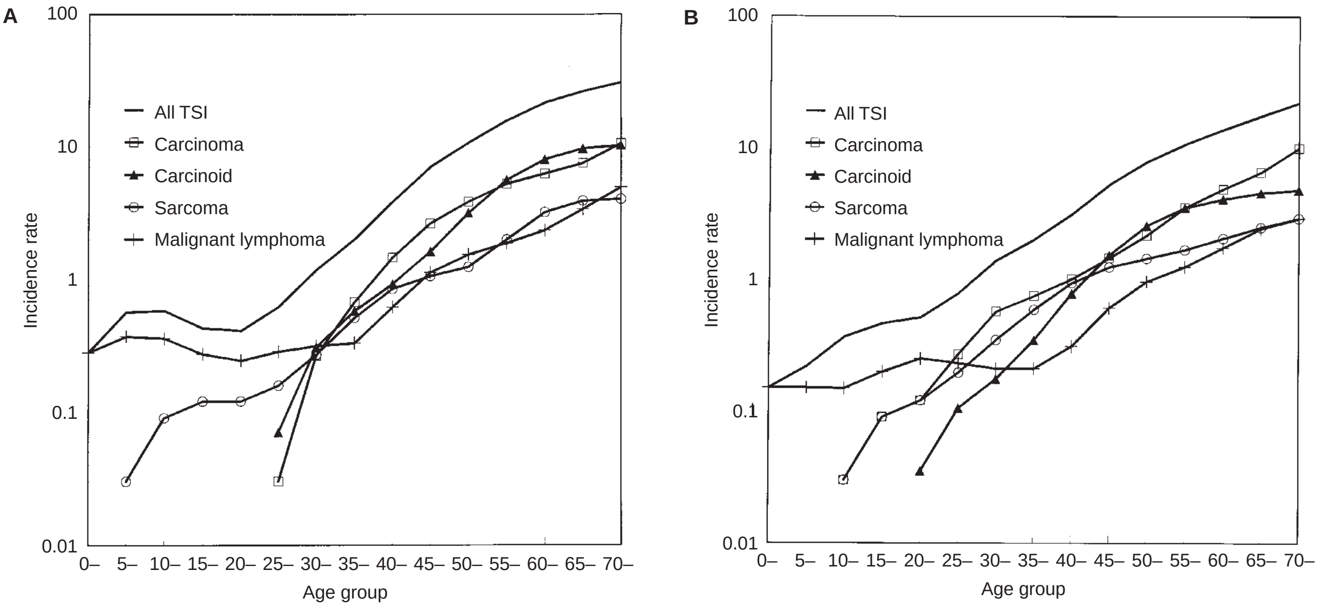

**Figure 1** Age-specific incidence rates for primary malignant tumours of the small intestine for males (**A**) and females (**B**) (cases per million), GDR 1976–1989 (1985 excluded)

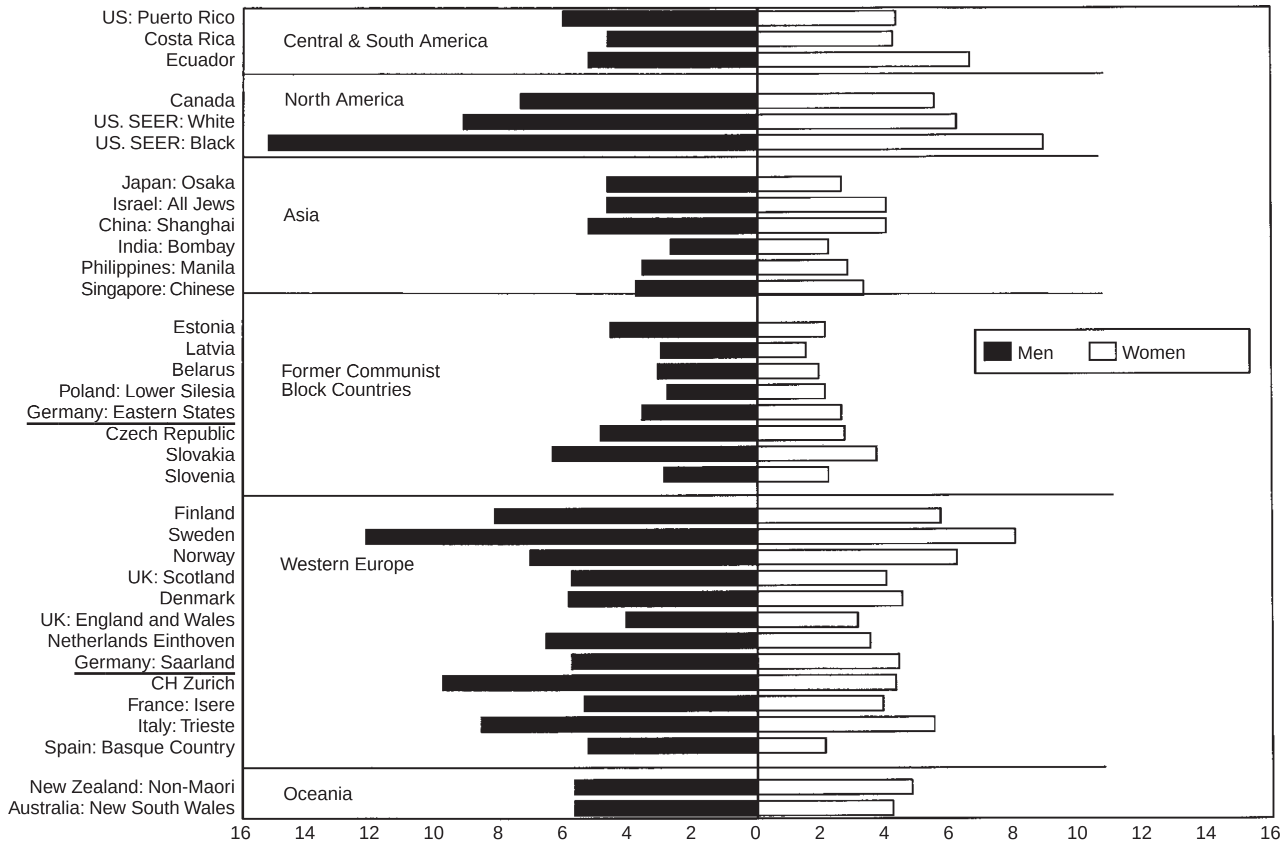

**Figure 2** Incidence rates for primary malignant tumours of the small intestine (malignant lymphoma excluded) in European Cancer Registries 1983–1992 (Parkin et al, 1992, 1997). Age-standardized incidence rates (WSR), truncated 0–74 years; cases per million (calendar year 1985 is excluded from the calculation of the incidence rates of the former GDR (1976–1989); Saarland Cancer Registry 1982–1993)

 *© 1999 Cancer Research Campaign*

Within the histopathologic subgroups, certain histologies are predominating. Leiomyosarcomas (63%) are the most frequent sarcomas followed by malignant schwannomas (7%). Adenocarcinomas (93%) are the most frequent carcinomas and non-Hodgkin's lymphomas (84%) are the most frequent malignant lymphomas.

Figure 1 presents age-specific incidence rates for all TSI and the four histopathological subgroups. The rates were quite low until the age of 30 years for all types, after which they rose sharply. For carcinomas, malignant lymphomas and sarcomas, the incidence rates continued to increase with each increasing 5-year age group, whereas that for carcinoids levelled off after the age of 54 years.

The sex-specific comparison of age-specific incidence rates of the histological subgroups from the former GDR shows that the higher carcinoma risk of males is mainly due to higher age-specific incidence rates between 40 and 64 years at age. The higher risk of carcinoids in males is mainly due to higher age-specific incidence rates between 55 and 74 years at age. Age-specific incidence rates for malignant lymphoma are continuously higher for males than for women. These differences are accentuated in the younger (0–10 years) and older age groups (70–74 years).

Table 3 presents the incidence rates for the four histopathological subgroups of TSI stratified by the origin of the tumours within the small intestine. The overall incidence of TSI continuously increases from the proximal part (duodenum) to the distal part of the small intestine (ileum). The histological subtypes show distinct topographical patterns within the small intestine: the incidence of carcinomas is highest in the duodenum; carcinoids and malignant lymphomas predominantly occur in the ileum.

The worldwide comparison shows that incidence rates tend to be lower in former communist block and Asian countries. Nations with western civilization (Western Europe and North America) tend to have the highest incidence rates. Within Western Europe, the incidence rates of the federal state of Saarland are represented in the middle range. Also, within the former communist block countries, incidence rates of the GDR are represented in the middle range (Figure 2).

### DISCUSSION

Compared to analyses of the USA (Weiss, 1987), we found that the risk for carcinoids in males is about 60% larger than in females. This risk difference is accentuated for the age range 55–74 years. The overall incidence rates of the German cancer registries were considerably lower than the rates from the USA, especially for men. In contrast to the USA, we did not find relevant increases of the incidence rates of all tumours of the small intestine combined or the histological subgroups for either sex over time. Analyses of cancer registry data from Canadian provincial registries by Gabos et al (1993) showed a small increasing trend for malignant lymphomas in both genders, although the incidence estimates were based on small numbers in comparison to the number of lymphoma cases in the GDR.

Several factors indicate that the aetiology within the group of TSI is heterogenous. Carcinomas, carcinoids, malignant lymphomas and sarcomas derive from different tissues and cell lines respectively. The majority of tumours of TSI that manifest before the age of 35 years are malignant lymphomas, whereas carcinoma and carcinoids prevail in the age group of 35 years and above. The histological subtypes of TSI show distinct topographical distributions within the small intestine. Predisposing diseases result in specific histological subtypes of TSI, e.g. malabsorptive diseases of the small intestine (gluten-sensitive enteropathy, idiopathic steatorrhoea and α-chain disease) and malignant lymphomas (Ellis, 1987), Crohn's disease and adenocarcinomas (Lightdale et al, 1975; Hoffman et al, 1977; Richards et al, 1989), familial polyposis syndromes and adenocarcinomas (Jagelman et al, 1988; Spigelman et al, 1989*a*, 1989*b*), immunosuppression or organ transplantation and malignant lymphomas (Penn, 1987).

Furthermore, dietary factors may increase the risk of TSI. In a correlational study, Lowenfels et al found that the amount of daily animal protein and animal fat consumption is positively associated with the risk of TSI (Lowenfels and Sonni, 1977). A recent case-control study by Chow et al (1993) showed that the frequent consumption of red meat or salt-cured/smoked foods is associated with two-to threefold increases of the risk of TSI. The ecological comparison of the meat consumption in the former GDR and the FRG until 1989 does not show a relevant difference in the per capita meat consumption per year (Bundesministerium für Landwirtschaft, 1980, 1997).

There are several factors of the study that may have affected our results. First, although the completeness of the analysed data sets of the two German cancer registries is about 90–95% (Parkin et al, 1997), the registration methods of the registries are not identical. For example, 40–50% of all new reports of cancer cases to the Saarland Cancer Registry come from pathologists only, whereas the majority of case reports to the registry of the GDR came from clinicians and pathologists. This difference might explain the lower incidence of malignant lymphoma of the small intestine in the federal state of Saarland because without additional clinical information, the reviewing pathologist cannot clearly decide whether a malignant lymphoma within the small intestine is a primary extranodal malignant lymphoma of the small intestine or a primary systemic malignant lymphoma with secondary involvement of the small intestine. The autopsy proportion in the GDR was substantially higher (25%) compared to West Germany (8%) (Becker, 1998). Although the higher proportion of autopsies in the GDR should result in higher incidence rates of TSI due to the discovery of asymptomatic TSI, the incidence rates of TSI in the GDR are lower than in the federal state of Saarland.

Second, within the periampullar region, adenocarcinomas can be misclassified as adenocarcinomas of the ductus of the bile (including the papilla of Vater), of the duodenum and of the pancreas (Jones et al, 1985). It is still unclear how much this potential misclassification contributes to the high proportion of carcinomas in the duodenum, which presents only about 4% of the length of the small intestine (Weiss, 1987). Also, tumours of the ileocaecum might be misclassified as colon or ileal tumours.

Third, malignant lymphomas may involve the small intestine primarily as extranodal malignant lymphomas with limited or regional lymph node involvement, or in connection with disseminated disease. It is often difficult to distinguish between primary and secondary involvement of the small intestine. The distinction becomes even more difficult when the tumour stage is advanced or when multiple sites within the gastrointestinal tract are involved (Ashley, 1988). Therefore, incidence estimates of malignant lymphomas of the small intestine must be evaluated cautiously.

Fourth, depending on the histopathological subgroup, 12–29% of cases with TSI are asymptomatic and discovered only coincidentally (Weiss, 1987; Matsuo et al, 1994). This finding indicates that we probably underestimate the incidence rate of TSI.

© 1999 Cancer Research Campaign 

### REFERENCES

Ashley SW and Wells SA Jr (1988) Tumors of the small intestine. *Semin Oncol* **15**: 116–128
Becker N and Wahrendorf J (1998) *Atlas of Cancer Mortality in the Federal Republic of Germany 1981–1990*, pp. 10–12. Springer-Verlag: Berlin
Brookes VS, Waterhouse JAH and Powell DJ (1968) Malignant lesions of the small intestine. A ten-year survey. *Br J Surg* **55**: 405–410
Bundesministerium für Landwirtschaft (1980) *Statistisches Jahrbuch über Ernährung, Landwirtschaft und Forsten*, pp. 112–113. Landwirtschaftsverlag Münster-Hiltrup: Münster
Bundesministerium für Landwirtschaft (1997) *Statistisches Jahrbuch über Ernährung, Landwirtschaft und Forsten*, pp. 146–147. Landwirtschaftsverlag Münster-Hiltrup: Münster
Chow WH, Linet MS, McLaughlin JK, Hsing AW, Co Chien HT and Blot WJ (1993) Risk factors for small intestine cancer. *Cancer Causes Control* **4**: 163–169
Ellis H (1987) Tumours of the small intestine. *Semin Oncol* **3**: 12–21
Gabos S, Berkel J, Band P, Robson D and Whittaker H (1993) Small bowel cancer in Western Canada. *Int J Epidemiol* **22**: 198–206
Hoffman JP, Taft DA, Wheelis RF and Walker JH (1977) Adenocarcinoma in regional enteritis of the small intestine. *Arch Surg* **112**: 606–611
WHO (1977) International Classification of Disease, 1975 revision. World Health Organization: Geneva
Jagelman DG, DeCosse JJ, Bussey HJR and The Leeds Castle Polyposis Group (1988) Upper gastrointestinal cancer in familial adenomatous polyposis. *Lancet* **1**: 1149–1151
Jones BA, Langer B, Taylor BR and Girotti M (1985) Periampullary tumors: which ones should be resected? *Am J Surg* **149**: 46–52
Lightdale CJ, Sternberg SS, Posner G and Sherlock P (1975) Carcinoma complicating Crohn's disease. *Am J Med* **59**: 262–268
Lowenfels AB and Sonni A (1977) Distribution of small bowel tumors. *Cancer Lett* **3**: 83–86
Matsuo S, Toshifumi E, Tsunoda T, Kanematsu T and Shinozaki T (1994) Small bowel tumors: an analysis of tumor-like lesions, benign and malignant neoplasms. *Eur J Surg Oncol* **20**: 47–51
Parkin DM, Muir CS, Whelan SL, Gao YT, Ferlay J and Powell J (eds) (1992) *Cancer in Five Continents*, Vol. VI. IARC Scientific Publication: Lyon
Parkin DM, Whelan SL, Ferlay J, Raymond L and Young J (eds) (1997) *Cancer in Five Continents*, Vol. VII. IARC Scientific Publication: Lyon
Penn I (1987) Cancers following cyclosporine therapy. *Transplantation* **43**: 32–35
Percy C, Van Holten V and Muir C (eds) (1990) *International Classification of Diseases for Oncology*, 2nd edn. World Health Organization: Geneva
Richards ME, Rickert RR and Nance FC (1989) Crohn's disease-associated carcinoma. *Ann Surg* **209**: 764–773
Selvin S (1996) *Statistical Analysis of Epidemiological Data*, 2nd edn, pp. 23–29. Oxford University Press: New York
Spigelman AD, Murday V and Phillips RKS (1989) Cancer and the Peutz-Jeghers syndrome. *Gut* **30**: 1588–1590
Spigelman AD, Williams CB, Talbot IC, Domizio P and Phillips RKS (1989) Upper gastrointestinal cancer in patients with familial adenomatous polyposis. *Lancet* **2**: 783–785
Statistisches Landesant Saarbrücken (1996) *Morbidität und Mortalität an Bösartigen Neubildungen im Saarland 1993* Sonderheft Nr. 186. Reihe des Statistischen Landesamtes Saarland: Saarbrücken
Weiss NS and Yang C-P (1987) Incidence of histologic types of cancer of the small intestine. *J Natl Cancer Inst* **78**: 653–656
WHO (1976) International Classification of Diseases for Oncology, 1st edn. World Health Organization: Geneva

*© 1999 Cancer Research Campaign*
